# Sotalol‐induced generalized and ocular myasthenia gravis

**DOI:** 10.1002/ccr3.2341

**Published:** 2019-07-28

**Authors:** Mohammadbagher Sharifkazemi, Farzad Ziya

**Affiliations:** ^1^ Department of Cardiology Shiraz University of Medical Sciences Shiraz Iran; ^2^ Department of Neurology Shiraz Central Hospital Shiraz Iran

**Keywords:** beta blockers, generalized myasthenia gravis, hypertrophic cardiomyopathy, ocular myasthenia gravis, sotalol

## Abstract

We present a patient with end‐stage hypertrophic cardiomyopathy who was suffering from ocular and generalized forms of myasthenia gravis as an uncommon neurological complication of sotalol. This case report warns clinicians to maintain caution over rare side effects of medication, which could be confused with the clinical symptoms of the underlying disease.

## 1 CASE PRESENTATION

**Figure 1:**
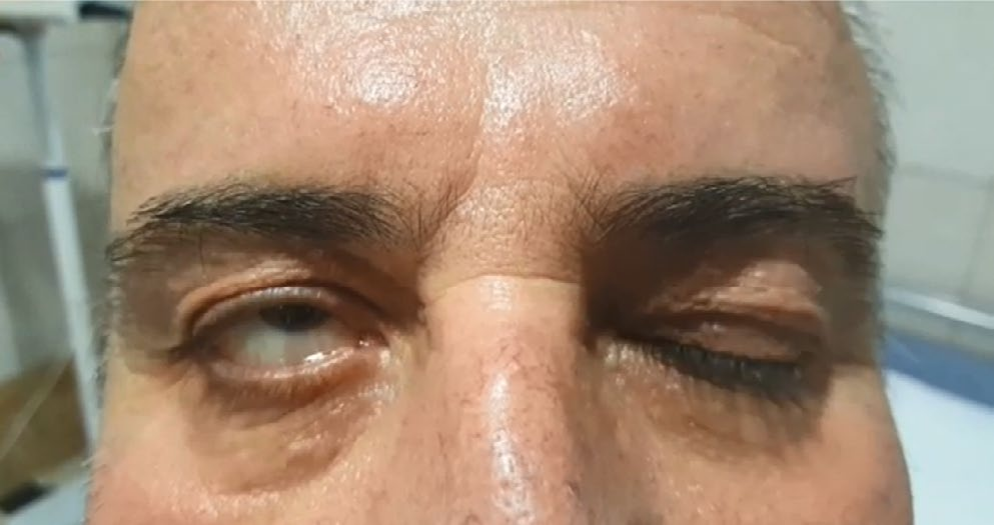
The upward rotation of the eyeballs during attempted eyelid closure; Bell's phenomenon

A patient with hypertrophic obstructive cardiomyopathy (HOCM), on sotalol, presented with signs and symptoms of ocular (Figure 1) and generalized myasthenia gravis (MG). Neostigmine and repetitive stimulation test was positive. EMG, NCV and antibodies against AChR and MuSK were negative. Work‐ups for thymus, thyroid, and autoimmune disorders were negative. Other neurological differential diagnoses were excluded. Single‐fiber EMG was unavailable. Sotalol was stopped and oral immunosuppressives plus IV IgG started. All neurological findings improved (Figure S1) during a period of 3 months. Sotalol, a beta blocker, was able to unmask, precipitate, or induce the MG syndrome.^1^, ^2^

## CONFLICT OF INTEREST

Authors declare no conflict of interest.

## AUTHORS' CONTRIBUTIONS

MS: prepared the manuscript and reviewed the literature. FZ: approved the final version of the manuscript.
